# miRPlant: an integrated tool for identification of plant miRNA from RNA sequencing data

**DOI:** 10.1186/1471-2105-15-275

**Published:** 2014-08-12

**Authors:** Jiyuan An, John Lai, Atul Sajjanhar, Melanie L Lehman, Colleen C Nelson

**Affiliations:** Australian Prostate Cancer Research Centre, Kragujevac, Queensland Australia; Princess Alexandra Hospital, Woolloongabba, Brisbane, Australia; Institute of Health & Biomedical Innovation (IHBI), Queensland University of Technology, Brisbane, Queensland Australia

**Keywords:** RNA-seq, miRNA, Plant small RNA, RNA secondary structure

## Abstract

**Background:**

Small RNA sequencing is commonly used to identify novel miRNAs and to determine their expression levels in plants. There are several miRNA identification tools for animals such as miRDeep, miRDeep2 and miRDeep*. miRDeep-P was developed to identify plant miRNA using miRDeep’s probabilistic model of miRNA biogenesis, but it depends on several third party tools and lacks a user-friendly interface. The objective of our miRPlant program is to predict novel plant miRNA, while providing a user-friendly interface with improved accuracy of prediction.

**Result:**

We have developed a user-friendly plant miRNA prediction tool called miRPlant. We show using 16 plant miRNA datasets from four different plant species that miRPlant has at least a 10% improvement in accuracy compared to miRDeep-P, which is the most popular plant miRNA prediction tool. Furthermore, miRPlant uses a Graphical User Interface for data input and output, and identified miRNA are shown with all RNAseq reads in a hairpin diagram.

**Conclusions:**

We have developed miRPlant which extends miRDeep* to various plant species by adopting suitable strategies to identify hairpin excision regions and hairpin structure filtering for plants. miRPlant does not require any third party tools such as mapping or RNA secondary structure prediction tools. miRPlant is also the first plant miRNA prediction tool that dynamically plots miRNA hairpin structure with small reads for identified novel miRNAs. This feature will enable biologists to visualize novel pre-miRNA structure and the location of small RNA reads relative to the hairpin. Moreover, miRPlant can be easily used by biologists with limited bioinformatics skills.

miRPlant and its manual are freely available at http://www.australianprostatecentre.org/research/software/mirplant or http://sourceforge.net/projects/mirplant/.

**Electronic supplementary material:**

The online version of this article (doi:10.1186/1471-2105-15-275) contains supplementary material, which is available to authorized users.

## Background

miRNA is a class of non-coding endogenous small RNA that post transcriptionally regulates target genes [1]. miRDeep-P [2] is one of the most commonly used computational plant miRNA identification tool, which is based on the miRDeep [3] algorithm.

The most challenging problem in identifying novel plant miRNA is to find a suitable genomic region as a miRNA precursor candidate (to test whether it forms hairpins) because the majority of precursor miRNA in plants are between 100-200 bp [4], which is much longer than those in animals. Approaches using a shorter miRNA precursor may result in false negatives if the miRNA is longer and more variable than the predicted precursor region. Conversely, using a longer candidate precursor region to test whether it forms a hairpin structure may result in a non-complimentary match for the mature miRNA within the candidate precursor miRNA. Thus, in miRPlant, after small RNA sequencing reads are mapped to the genome, genomic regions around mapped reads are extended by 200 bp to determine whether they form hairpin structures. To ensure detection of short plant miRNA, we also scan 100 bp regions to see if we can detect a hairpin. This strategy can detect bona fide miRNAs that would otherwise be missed if only the longer (200 bp) precursor candidate length was used.

**Figure 1:**
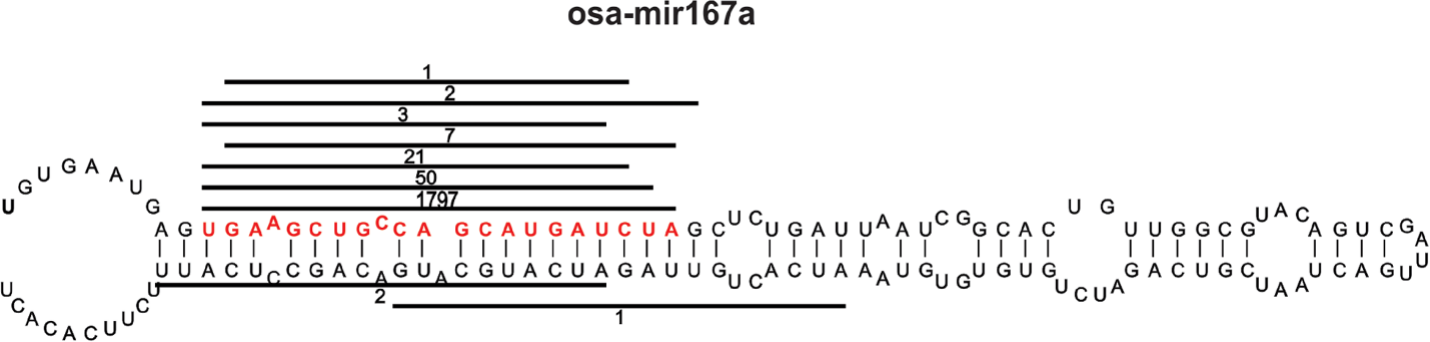
**Output display of predicted miRNA.** The read location and number of reads are shown relative to the precursor hairpin structure. The red sequence represents the mature miRNA.

The strategy for determining the precursor region is different between miRDeep-P and miRPlant. miRDeep-P determines the precursor region based on the genomic region having overlapping reads, while miRPlant determines a precursor region based on the mature miRNA region (or highest expressed read). The latter strategy can reduce the number of false negative results [5, 6], as it guarantees that the mature miRNA is located at the end of one arm of the stem loop.It is important that biologists with basic computer skills can easily use RNAseq tools in order to broaden research within this field. Thus, miRPlant was developed using the platform independent computer language Java. A Graphical User Interface (GUI) is employed whereby a complete pipeline analysis of raw data input is achieved in a few clicks of buttons: (.fastq files) - > mapping (.bam files) - > miRNA identification, expression, and secondary structure display - > mRNA target prediction. To further streamline accessibility of miRPlant, the tool does not require any third party tool. miRPlant also has a detailed but concise data output display that can be exported for publication in different file formats such as eps, pdf and svg (Figure 1). miRPlant images are generated dynamically.

## Implementation

miRPlant operations can be divided into the following stages:

i. filter out reads if their length is out of the 10-23 bp range, or which have a read-quality below the criteria that is set by user.
ii. aggregate exact reads into one.
iii. map aggregated reads to the genome reference without mismatch. miRPlant uses the Java-coded bowtie [7] alignment algorithm. BAM format is used to store mapped reads. Please note that the attribute “XS” in the BAM file is used to record the copy number of the read as introduced by miRDeep*.
iv. gather sequences in the reference genome flanking the RNAseq read (precursor miRNA region) to determine whether the genomic region forms a hairpin structure using the RNA secondary structure algorithm [8].
v. use the miRDeep model to calculate the score for each predicted miRNA to measure the strength of the prediction. A higher score equates to a higher probability that the predicted miRNA is true.

**Figure 2:**
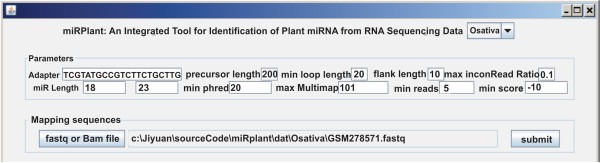
**Parameter settings for miRPlant.** Adapter sequences need to be replaced as appropriate. Data processing by miRPlant depends on the extension of the input file. Mapping and identification is performed if the input file extension is “.fastq” or “.fa”. Only identification is performed if the file extension is “.bam”. Output “.result” files are shown after clicking “submit”.

The miRPlant interface enables users to customize parameters since different plant species may have different miRNA biogenesis [2] (Figure 2). The default precursor miRNA length is set to 200 bp. Here the precursor length represents the length between the mature miRNA and the mature star miRNA; the two flanking sequences are excluded. miRPlant generates six output files similar to miRDeep*. Since the precursor length of plant miRNA is much longer than that of animals, the distance between the mature miRNA and mature star miRNA may be very long, which may result in the formation of an internal loop. Therefore, miRPlant allows for internal loops. The default minimum loop (including the distance from loop ends to the mature or star mature miRNA) size is 25 bp. In predicting mature miRNA, miRPlant requires less than 10% (max inconRead Ratio option in GUI) of reads falling out of the predicted miRNA and star mature miRNA sequence. In miRDeep, RNAseq reads in the loop are counted as being consistent, but plant miRNA have very long loops. Thus, we exclude reads located within the loop region. The other parameters are the same as with miRDeep*.

## Results and discussion

miRPlant has been tested on two rice datasets [9]. Both miRPlant and miRDeep-P employ the miRDeep score calculation, with miRPlant having better performance than miRDeep-P (Table 1), largely because miRPlant uses a flexible method to form the precursor candidates from the genomic region surrounding RNAseq reads. We set a minimum score of four when using miRPlant. A detailed summary of results can be found in Additional file 1 using GEO access number GSM278571 and GSM278572 for the RNAseq datasets.

**Table 1:** **Comparison table**

	Rice (GSM278571)		Rice (GSM278572)	
Tool	miRDP	miRPlant	miRDP	miRPlant
**Precision**	0.82(31/38)	0.95(36/38)	0.7 (44/63)	0.83 (52/63)
**Recall**	0.22 (31/144)	0.25 (36/144)	0.24 (44/181)	0.29 (52/181)

Precision = known MiR/predicted MiR Recall = known MiR/total known MiR.

To further confirm the advantaged of miRPlant, we have extended this analysis to three more species (Arabidopsis thaliana, Medicago truncatula and Prunus persica) comprising 16 small RNA sequencing datasets (Detailed information in Additional file 2). To compare the two tools, we rank the predicted miRNAs in descending order of score for each tool, and then take the top 100 miRNAs from miRPlant and miRDeep-P for our comparison. We show that miRPlant consistently outperforms these other tools in all samples (Table 2, Additional files 3 and 4).

**Table 2:** **Comparison table** (**ATH**, **MTR**, **PPE**)

	A. thaliana (Number of known miRNA: 121)		M. truncatula (Number of known miRNA: 196)		P. persica (Number of known miRNAs: 75)	
Tool	miRDP	miRPlant	miRDP	miRPlant	miRDP	miRPlant
**Precision**	0.405	0.51	0.22	0.66	0.2	0.55
**Recall**	0.35	0.65	0.10	0.325	0.29	0.65

Precision = known MiR/predicted MiR Recall = known MiR/total known MiR.

## Conclusions

miRPlant is modelled off miRDeep* [5] for use with plant small RNA sequencing data. We have integrated all third party tools such as genomic mapping and RNA secondary structure prediction [8] into a Java library, which is seamlessly integrated into miRPlant.

## Availability and requirements

**Project name:** miRPlant.

**Project home page:**http://www.australianprostatecentre.org/research/software/mirplant.

**Operating system (s):** Windows, Linux, Mac OS.

**Programming language:** Java.

**Other requirements:** JRE.

**License:** GNU General Public License.

**Any restrictions to use by non-academics:** None.

## Electronic supplementary material

Additional file 1: **List of all identified miRNAs from two rice small RNAseq data.** (XLSX 337 KB)

Additional file 2: **Small RNA sequencing data details.** (DOCX 44 KB)

Additional file 3: **Detailed result of miRPlant prediction.** (XLSX 13 MB)

Additional file 4: **Detailed result of miRDeep-P prediction.** (XLSX 541 KB)

## References

[1] Pritchard CC, Cheng HH, Tewari M (2012). MicroRNA profiling: approaches and considerations. Nat Rev Genet.

[2] Yang X, Li L (2011). miRDeep-P: a computational tool for analyzing the microRNA transcriptome in plants. Bioinformatics.

[3] Friedlander MR, Chen W, Adamidi C, Maaskola J, Einspanier R, Knespel S, Rajewsky N (2008). Discovering microRNAs from deep sequencing data using miRDeep. Nat Biotechnol.

[4] Meyers BC, Axtell MJ, Bartel B, Bartel DP, Baulcombe D, Bowman JL, Cao X, Carrington JC, Chen X, Green PJ, Griffithsnes S, Jacobsen SE, Mallory AC, Martienssen RA, Poethig RS, Qi Y, Vaucheret H, Voinnet O, Watanabe Y, Weigel D, Zhu JK (2008). Criteria for annotation of plant MicroRNAs. Plant cell.

[5] An J, Lai J, Lehman ML, Nelson CC (2013). miRDeep*: an integrated application tool for miRNA identification from RNA sequencing data. Nucleic Acids Res.

[6] Friedlander MR, Mackowiak SD, Li N, Chen W, Rajewsky N (2012). miRDeep2 accurately identifies known and hundreds of novel microRNA genes in seven animal clades. Nucleic Acids Res.

[7] Langmead B, Trapnell C, Pop M, Salzberg SL (2009). Ultrafast and memory-efficient alignment of short DNA sequences to the human genome. Genome Biol.

[8] Hofacker IL (2003). Vienna RNA secondary structure server. Nucleic Acids Res.

[9] Zhu QH, Spriggs A, Matthew L, Fan L, Kennedy G, Gubler F, Helliwell C (2008). A diverse set of microRNAs and microRNA-like small RNAs in developing rice grains. Genome Res.

