# Supplementary material for: miRPlant: an integrated tool for identification of plant miRNA from RNA sequencing data
Source: BMC Bioinformatics. 2014 Aug 12;15(1):275. doi: 10.1186/1471-2105-15-275 (PMC4141084; doi:10.1186/1471-2105-15-275)
Supplement: Supplementary file 2 — Additional file 2: Small RNA sequencing data details. (DOCX 44 KB) [file 12859_2014_6544_MOESM2_ESM.docx]

￼small RNA sequencing data details

Species Arabidopsis thaliana (8 samples)

Access ID in NCBI (http://www.ncbi.nlm.nih.gov/)

SRR275588 SRR051927 SRX065858 SRX065854 SRX065857 SRX107308 SRX107309 SRX107310

Medicago truncatula (4 samples)

Access ID in NCBI (http://www.ncbi.nlm.nih.gov/)

GSM769272 GSM769273 GSM769274 GSM769277

Prunus persica (4 samples)

Access ID in NCBI (http://www.ncbi.nlm.nih.gov/)

GSM944971 GSM944972 GSM944973 GSM944974
